# Fly-Cutting Processing of Micro-Triangular Pyramid Arrays and Synchronous Micro-Scrap Removal

**DOI:** 10.3390/mi15050655

**Published:** 2024-05-16

**Authors:** Jiashun Gao, Zhilong Xu, Yu Lei, Su Huang

**Affiliations:** 1 Xiamen Ocean Vocational College, Xiamen 361000, China; gaoshunjia@163.com (J.G.); huangsuxmoc@163.com (S.H.); 2 School of Marine Engineering, Jimei University, Xiamen 361000, China; 3 School of Marine Equipment and Mechanical Engineering, Jimei University, Xiamen 361000, China; 4 Institute of Manufacturing Engineering, Huaqiao University, Xiamen 361000, China; 13886568044@163.com

**Keywords:** micro-triangular pyramid array, micro-scrap, scratch, fly-cutting, vertical orientation

## Abstract

Many micro-scraps are generated when a micro-triangular pyramid array (MTPA) is machined by the fly-cutting method. Micro-scraps are generally not removed quickly enough; therefore, these residual micro-scraps participate in the cutting process again, scratching the workpiece surface and accelerating diamond tool wear. To remove micro-scraps rapidly, a fly-cutting method to produce MTPAs on vertically oriented working surfaces was developed during this study. The results show that an MTPA produced by fly cutting on a vertical workpiece had a clearly outlined structure, high dimensional accuracy, and a low surface roughness. There was no micro-scrap residue on the workpiece surface and the diamond tool wear was small. The cutting inlet edges had no burrs, and the cutting outlet edges had only a small number of burrs. This method of fly cutting MTPAs on vertically oriented working surfaces provides a foundation for the development of high-precision micro-triangular pyramid optical elements.

## 1. Introduction

Micro-triangular pyramid (MTP) molds of copper, aluminum, and other metals are key parts of the manufacturing processes of precision optical components, such as reflective sheeting and light-trapping film. The optical functional surfaces of these components are composed of micro-triangular pyramid arrays (MTPAs) with edge lengths less than 100 μm. At present, the primary MTPA manufacturing methods include laser-induced etching [1], chemical etching [2], electrical discharge forming machining [3], and mechanical machining [4,5]. Among these, laser-induced etching and chemical etching have produced good results during the processing of silicon-based materials, though they are rarely used to process plastic metals, such as copper and aluminum [1]. Electrical discharge forming machining can be used to process brittle and hard metals; however, copper electrodes must be prepared in advance, and this is carried out by mechanical machining [6]. Therefore, mechanical machining is the primary processing method used for MTP molds of copper, aluminum, and other metals; the specific machining methods include diamond micro-chiseling [4], grinding [3], planing [7], and fly-cutting [5,8].

The diamond micro-chiseling method can be used to process micro-cube arrays with complex shapes [9]; this procedure has stringent machine tool requirements and a complex tool-setting process. The machining efficiency of forming microstructures with a grinding wheel is high, but the abrasive particles fall off the wheels during grinding, thereby causing damage to the machined surfaces [10,11]. V-shaped diamond planing tools allow for the machining of MTPAs with slow cutting speeds, large cutting forces, large material plastic deformation, and the generation of burrs on the MTP edges [7,12]. The fly-cutting method is widely used to machine MTPAs because it uses rapid cutting speeds and produces accurate machining results [13]. At present, when an MTPA is processed using the fly-cutting method, the workpiece is placed horizontally on the workbench while the tool rotates quickly above it. Through cooperative motion of the workbench and the tool, the MTPA is generated on the upper surface of the workpiece [14].

Because the fly-cutting method is a discontinuous cutting method, it inevitably produces many micro-scraps with thicknesses of approximately 2 mm. Some of the micro-scraps that remain on the MTP surfaces are squeezed and scratched by the diamond tool; thus, they can easily produce scratches on the surfaces of the MTPAs, and can even cause the edge or tip of the diamond tool to break [15,16]. Therefore, it is necessary to continuously remove micro-scraps when using the fly-cutting method to process MTPAs. The current micro-scrap removal methods include synchronous removal during machining and ultrasonic cleaning after machining. Some scholars have used coolant to remove micro-scraps during processing; most of the micro-scraps could be removed during the machining process when this method was used, but some micro-scraps remained on the MTPA surfaces [5,9,17]. Some scholars used ultrasonic cleaning to remove micro-scraps after machining; this method produced good micro-scrap removal effects, but micro-jets generated by ultrasonic cavitation could easily damage the MTPA surfaces, thereby adversely affecting the workpiece surface quality [18,19].

**Figure 1:**
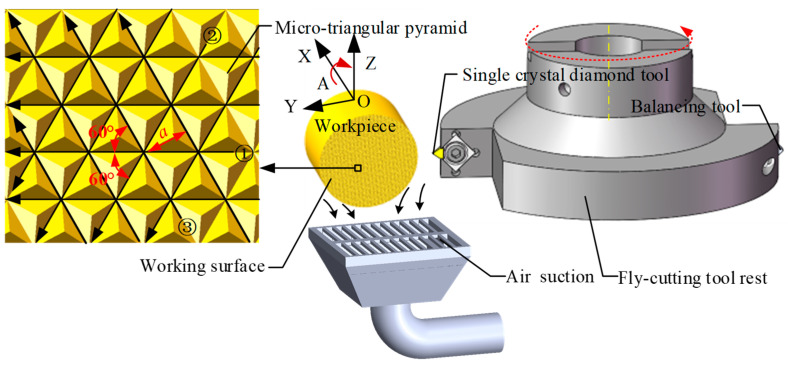
MTPA fly-cutting method with a vertically oriented working surface. V-shaped grooves were cut in three directions, and the distance between V-shaped grooves cut in the same direction, a, was 100 μm. The angle between two adjacent cutting directions was 60°.

In the current study, an MTPA fly-cutting method, in which the working surface is oriented vertically, was developed so that micro-scraps could be removed quickly. This new method is illustrated in Figure 1. When the working surface is vertically oriented, the combination of gravity and compressed air causes the micro-scraps produced by the fly-cutting method to easily slide off the MTPA surface; then they are discharged from the working area by a suction channel. Theoretically, this method can prevent micro-scraps from remaining on the surface of the MTPA and can thus also prevent extrusion and scratching between the diamond tool and the micro-scraps. Diamond tool edge or tip breakage and subsequent scratching of the MTP surfaces can also be avoided. When the fly-cutting process is complete, no micro-scraps remain on the surface of the workpiece. The workpiece does not require ultrasonic cleaning but must only be soaked in alcohol; thus, the MTPA surface damage caused by micro-jet impacts during ultrasonic cleaning can be prevented. To verify the feasibility of this method, theoretical analysis and experimental research are carried out.

## 2. Experiment Preparations

### 2.1. Geometric Parameters of the MTPs and the Diamond Tool

**Figure 2:**
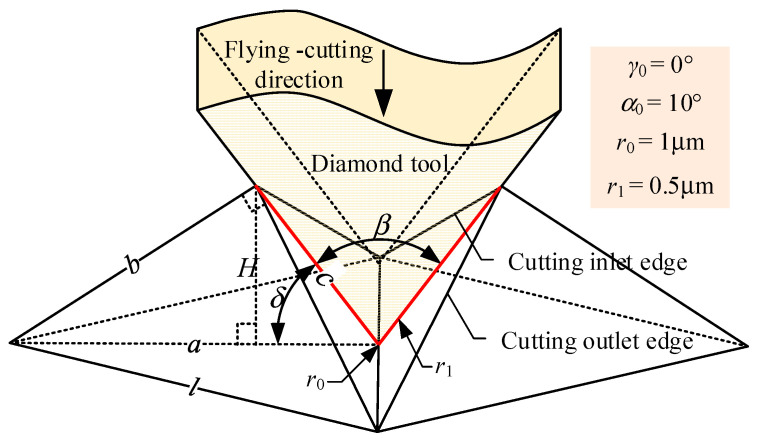
Geometric parameters of the MTP and the diamond tool.

The three sides of the MTP used in this study were isosceles right triangles, and the bottom surface was an equilateral triangle with a height, *a*, of 100 μm, as shown in Figure 2. The side length, *l*, of the bottom surface was 115.47 μm; this value was used in Equations (1)–(5) to determine that the side edge length, *b*, was 81.64 μm, the side height, *c*, was 57.75 μm, and the total height, *H*, of the MTP was 47.16 μm. The angle between a side and the bottom surface, *δ*, was 54.8°, and the angle between two opposite sides, *β*, was 70.5°.

(1) $$a=\sqrt{3}l/2,$$

(2) $$b=\sqrt{2}l/2,$$

(3) δ=arcsin (b/a),

(4) c=a·cosδ,

(5) β=π−2δ,

According to the MTP geometry, a single-crystal diamond molding grain was designed and welded onto an SWHT1204 fly-cutting tool. The two cutting edges of the grain were symmetrically distributed, and the grain had an included angle, *β*, of 70.5°. The front angle of the tool, *γ*_0_, was 0°, the back angle, α_0_, was 10°, the radius of the tool nose, *r*_0_, was 1 μm, and the radius of the cutting edge, *r*_1_, was 0.5 μm, as shown in Figure 2.

### 2.2. Machine Tool and Workpiece Material

**Figure 3:**
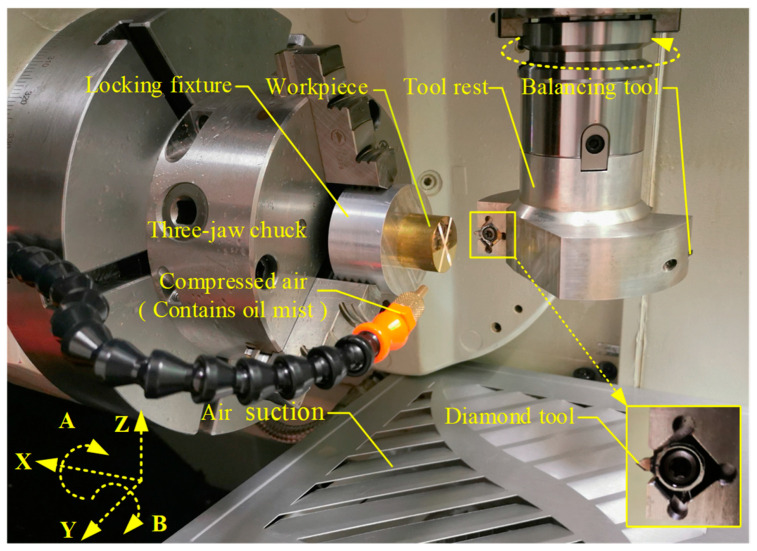
MTPA fly-cutting process conducted with a five-axis machine tool.

The MTPA fly-cutting process was conducted on a WN-5V250 five-axis machine tool. The machine tool used three moving axes and two rotating axes to collaboratively control the relative positions of the diamond tool and the workpiece. It used a machine spindle to rotate the diamond tool rapidly, thereby achieving fly-cutting of the MTPA, as shown in Figure 3. The resolution of the three linear axes was 0.1 μm and the resolution of the rotating axes (A) was 0.001°. The air eddies generated by the rapid rotation of the fly-cutting components caused some of the micro-scraps to re-adhere to the workpiece surface. To accelerate the falling of the micro-scraps, an air suction device was installed. Copper and aluminum are relatively soft with respect to steel and have good adhesion characteristics. To prevent sticking during the processing, an oil mist that produced lubrication, cooling, and anti-oxidation effects in the cutting area was added to compressed air. During processing, the compressed air that contained this oil mist was sprayed into the processing area to facilitate lubrication and cooling and to prevent oxidation. In addition, to prevent thermal deformation of the workpiece material and the machine tool, an air-conditioning cooling system was used to maintain the temperature of the cutting environment at 25 ± 0.2 °C.

**Figure 4:**
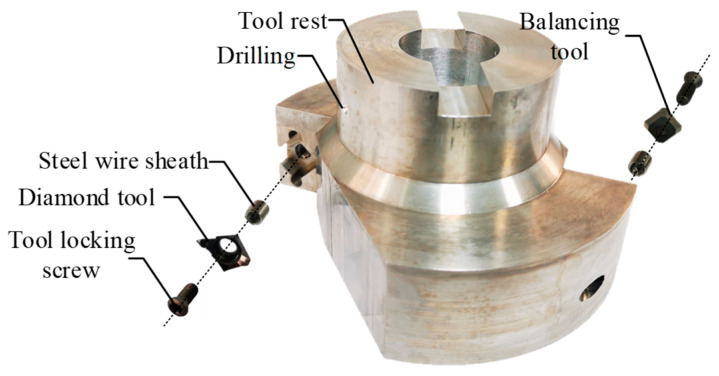
Unassembled fly-cutter component.

To prevent tool rod jitter and resonance during the high-speed fly-cutting process, the fly-cutter components were designed, manufactured, and installed, as shown in Figure 4. The 6061-aluminum alloy was used as the tool rest material to reduce the fly-cutter component weight. To facilitate dynamic balance adjustments, the tool rest was provided with two center-symmetric tool mounting slots; a diamond tool was installed in one of these slots, while a balancing tool was installed in the other. Frequent tool replacement tends to cause threaded hole failure in aluminum tool rests. Threaded holes are difficult to repair; thus, if they fail, the tool rest must be replaced. Therefore, steel wire sheaths with anti-loosening functions were used as substitute for threaded holes. When the threads became damaged, therefore, a new tool rest was not needed; rather, it was only necessary to replace the steel wire sheaths. Before machining was performed, the dynamic balance of the fly-cutter components was calibrated by the drilling method, and the location of the drilling hole is shown in Figure 4. After the fly-cutter components were assembled, the tool nose rotation diameter was 100 mm.

**Figure 5:**
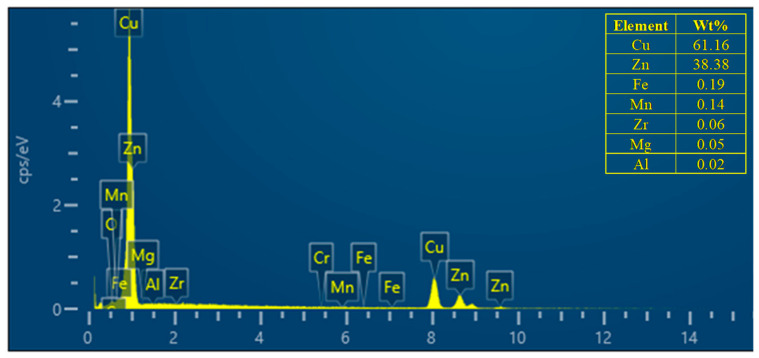
EDS results for the chemical composition of the workpiece material.

**Figure 6:**
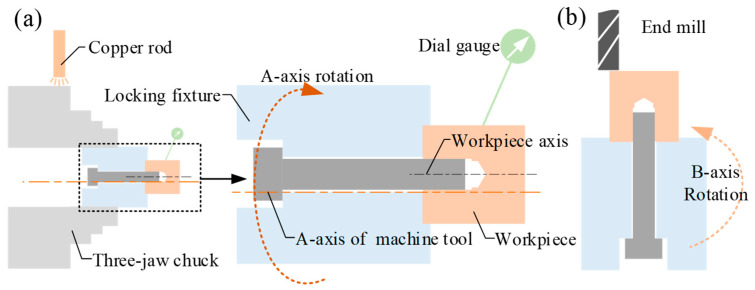
Preparation of the workpiece for machining: (**a**) correction of the circular run-out of the outer circular surface of the workpiece and (**b**) milling of the working surface.

The workpiece was a brass bar with a diameter of 30 mm and a length of 20 mm. Its material grade was C28000. Measured energy dispersive spectroscopy (EDS) results (obtained by a Crossbeam 550 Oxford Xplore30 machine) showed that the chemical composition of the workpiece met the requirements of the ASTMB36/B36M standard [20]; its Cu content was 61.16% and its Zn content was 38.38%, as shown in Figure 5. The dynamic friction coefficient between the workpiece and the single-crystal diamond tool was 0.18 [7], and the possibility of direct micro-scrap adherence to the tool was low. To facilitate clamping, machining was performed to place M8 thread holes in the bottom of the workpiece, as shown in Figure 6a.

### 2.3. Preparation of the Workpiece for Machining

To prevent the three-jaw chuck from deforming the workpiece, the workpiece was locked, using a screw, into a clamping groove with a diameter of 30 mm and a length of 5 mm on the end face of the locking fixture. Then, the locking fixture was clamped by the three-jaw chuck. To ensure that the axis of the workpiece and the A-axis of the machine tool could overlap, the A-axis rotated slowly while a dial gauge was used to calibrate the outer circular surface of the workpiece. If the circular run-out exceeded 1 μm, the three-jaw chuck was tapped with a copper rod until the circular run-out met the requirements, as shown in Figure 6a. Before machining the MTPA, the upper surface of the workpiece was made horizontal by rotating the B-axis of the machine tool while the working surface was milled with an end mill, as shown in Figure 6b. After the milling was complete, the burrs on the working surface were removed with an oilstone to prevent them from damaging the diamond tool.

## 3. Experimental Methods

### 3.1. Tool Installation, Tool Path, and Cutting Parameters

The investigation regarding the tool installation method is discussed next. The diamond tool was installed on the fly-cutting tool rest, and the fly-cutting tool rest and the tool holder were locked into and installed on the machine spindle. The spindle drove the tool rotation, and thus also the diamond tool nose rotation, and thereby cut the V-shaped grooves. Since there was only one diamond tool on the fly-cutting tool rest, one cut was made in the workpiece during each spindle rotation.

V-shaped grooves were cut in three directions, and the distance between V-shaped grooves cut in the same direction, a, was 100 μm. The angle between two adjacent cutting directions was 60°, as shown in Figure 1. When the V-groove cutting in one direction was complete, the A-axis rotated clockwise by 60° to cut the V-groove in the second direction. Finally, the A-axis rotated counterclockwise by 120° to cut the V-shaped groove in the third direction.

**Figure 7:**
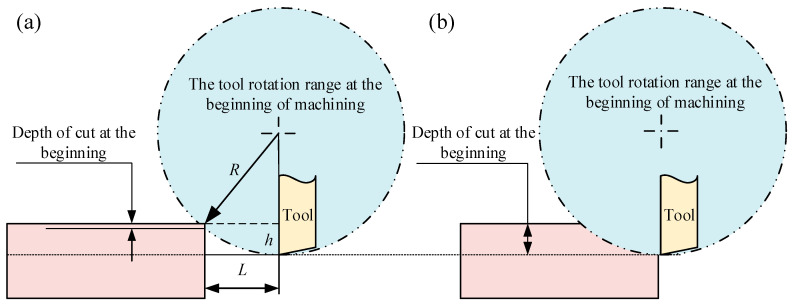
Tool and workpiece positions at the start of machining: (**a**) the distance between the tool and the workpiece is equal to *L* and (**b**) there is no distance between the tool and the workpiece.

The total height of the MTPA, *H*, was 47.16 μm. To ensure the integrity of the MTP machining, the V-groove cutting depth, *h*, was 60 μm. To ensure that the cutting depth was the same during each rotation, a certain distance, *L*, between the tool and the workpiece had to be maintained, as shown in Figure 7a. If this was not done, the cutting depth produced during the first rotation of the tool would be too large and would result in tool nose damage, as shown in Figure 7b. According to Equation (6), this distance, *L*, should be greater than or equal to 2.45 mm before processing to ensure uniformity between the cutting depths of different rotations:

(6) $$L=\sqrt{R^{2}-{\left(R-h\right)}^{2}}.$$

In Equation (6), R represents the turning radius of the tool nose, *h* is the depth of the V-shaped groove, and *L* is the distance between the tool and the workpiece before machining.

**Figure 8:**
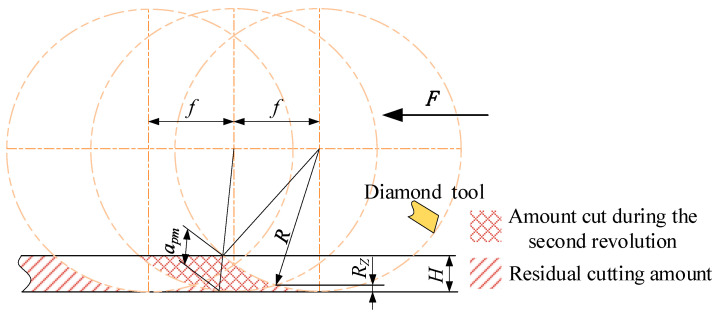
Schematic diagram of the cutting parameters.

The difference between the theoretical cutting depth, *h*, and the actual maximum cutting depth, *a_pm_*, was significant. Figure 8 presents a schematic diagram of the cutting parameters of the tool for three revolutions. Because the feed amount, *f*, was much smaller than the radius of the tool nose, *R*, for a clearer expression, the actual vortex-line track of the tool nose was simplified into three double-point scribe-line circular tracks. The relationships between the rotational speed, *n*, the feed rate, *F*, the feed amount, *f*, the maximum surface roughness, *R_Z_*, the cutting velocity of the tool nose, *v*, and the actual maximum cutting depth, *a_pm_*, are shown in Equations (7)–(10):

(7) f=F/n,

(8) v=2πrn,

(9) $$R_{Z}=R-\sqrt{R^{2}-{\frac{f}{4}}^{2}},$$

(10) $$a_{pm}=R-\sqrt{{\left(R-h\right)}^{2}+{\left(\sqrt{R^{2}-{\left(R-h\right)}^{2}}-\frac{f}{2}\right)}^{2}},$$

During machining, *n* = 1000 r/min and *F* = 60 mm/min; thus, *v* = 5.23 m/s, *f* = 60 μm/r, *R_Z_* = 9 nm, and *a_pm_* is approximately equal to 2 μm. Theoretically, V-shaped grooves that meet the surface quality requirements can be machined using these cutting parameters, which are listed in Table 1.

### 3.2. Tool Setting Process

**Figure 9:**
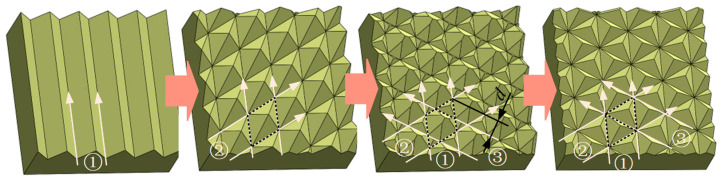
Tool setting procedure.

To ensure that the cutting paths in all three directions would intersect at a single point, the trial-cutting method was adopted during the tool setting process, as shown in Figure 9. Two workpieces were prepared for this method; one was a trial-cutting workpiece and the other was the final workpiece. The trial-cutting procedure is described next. First, to prevent the tool from wearing too quickly during the trial-cutting process, a small number of MTPAs were cut into the trial-cutting workpiece. Then, the trial-cutting workpiece was placed under a confocal laser microscope for observation to determine whether the bottoms of the V-shaped slots in all three directions intersected at a single point. If they did not intersect, the vertical distance between the intersection points of the bottom of the V-shaped groove in the third direction and the bottoms of the V-shaped grooves in the first two directions, *d*, was measured. Next, *d* was adjusted within the trial-cutting program so that the V-shaped slot in the third direction was offset by d, and a small number of MTPAs were cut again. This process was repeated until the V-shaped grooves in all three directions intersected at a single point. Finally, the final trial-cutting deviation, d, was adjusted in the final machining program and the MTPA machining could be completed.

## 4. Results and Discussion

### 4.1. MTP Size and Surface Roughness

**Figure 10:**
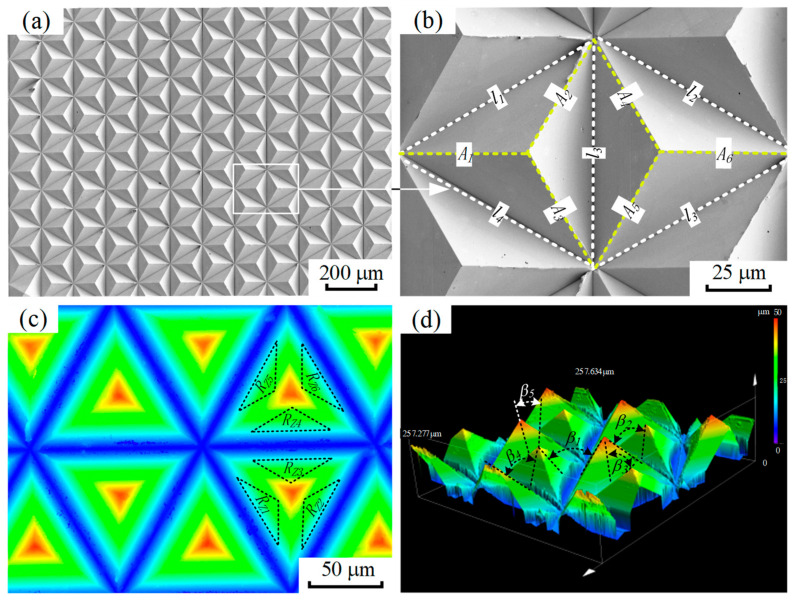
MTP size and surface roughness measurements: (**a**) SEM results for the MTPA, (**b**) SEM measurements of *l* and *A*, (**c**) CLSM measurements of *R_Z_*, and (**d**) CLSM measurements of *β*.

The MTP size and surface roughness directly affect the optical functions of optical elements; the MTP size affects the propagation path of light, while the surface roughness affects the reflectivity of light. The dimensions of the MTP include the bottom triangle side length, the angle between opposite sides, and the edge straightness. To prevent scratching of the workpiece surface during measurement, a non-contact measurement method was adopted. Scanning electron microscopy (SEM) with a Crossbeam 550 machine was used to detect the bottom triangle side length, *l*, and the edge straightness, *A*, of the MTP, as shown in Figure 10a,b. Confocal laser scanning microscopy (CLSM) performed by an OLS5100 machine with a violet light source was used to measure the surface roughness, *R_Z_*, and the angle between opposite sides, *β*, as shown in Figure 10c,d. Three regions, each containing two complete MTPs, were randomly selected for measurement. In each region, five *l* values, six *A* values, five *β* values, and six *R_Z_* values were measured, as shown by the dashed lines in Figure 10b–d.

The test results are presented in Table 2. The average bottom triangle side length of the MTPA was 114.86 μm, while the standard value was 115.47 μm. The smaller average bottom triangle side length may have been caused by the influence of the radius of the tool nose on the bottom shape of the V-shaped groove. Since the deviation was only 0.53%, the MTPA demonstrated good consistency with respect to the bottom triangle side length and a high degree of intersection between the V-shaped grooves in all three directions. The actual angle between opposite sides was 70.6 degrees, while the standard value was 70.5 degrees; thus, the deviation was 0.1% with respect to the standard value. The large average angle between opposite sides may have been caused by micro-plastic deformation of the sides of the V-shaped grooves produced by tool-induced extrusion of the machined surface during the fly-cutting process. The average edge straightness of the MTPA was 2.37 μm greater than the standard value of 1 μm. The large edge straightness was caused by residual burrs on some of the cutting outlet edges, as shown in Figure 10b. The straightness of the cutting inlet edges was good, especially when the V-shaped grooves were cut in the third direction; the residual burrs on the cutting outlet edges were large, however. The surface roughness can reflect the machining quality of the MTPA sides better than the linear roughness. The average surface roughness of the MTPA sides was 19.1 nm, and thus much smaller than the wavelengths of visible light.

### 4.2. Remaining Micro-Scraps

**Figure 11:**
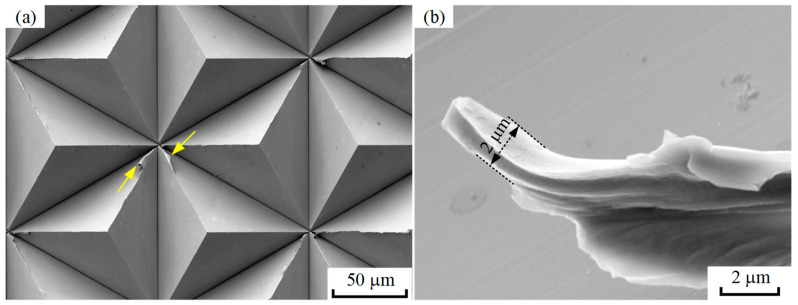
SEM images of MTPs and micro-scraps: (**a**) MTP unit (the yellow arrows indicate burrs on the cutting outlet edges) and (**b**) micro-scraps with a maximum thickness of approximately 2 mm.

The action of the cutting force produces plastic deformation of the material during cutting, which results in scraps [21,22,23,24]. According to Equations (7)–(9), when the rotational speed is 1000 r/min and the feed rate is 60 mm/min, at least 1000 micro-scraps with thicknesses of approximately 2 μm will be generated every minute. The workpiece material used in this study was soft, and was therefore conducive to micro-scrap adherence to the MTPA surface. If the micro-scraps participated in the cutting process again, they could easily be squeezed between the cutting edge of the tool and the workpiece surface; these micro-scraps could then scratch the MTPA surface or even cause the tool to break. The SEM observation results indicate that no residual micro-scraps or side scratches were present on the MTP surfaces, as shown in Figure 11a. Micro-scraps were collected from the filter of the suction device and observed using SEM. It was found that they had thicknesses of approximately 2 μm, as shown in Figure 11b; this value was near that of the maximum cutting depth.

**Figure 12:**
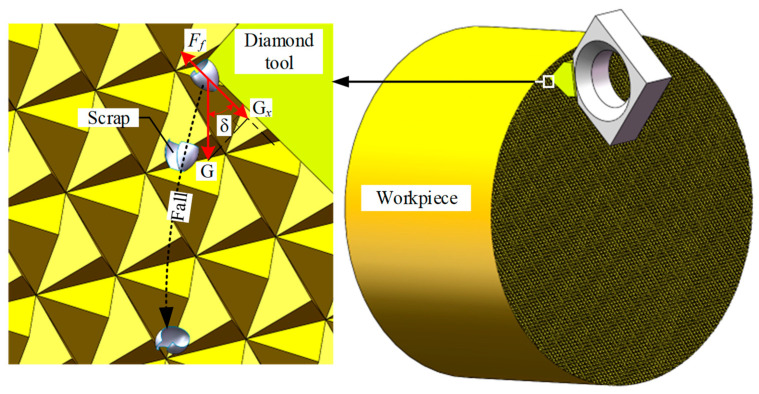
Process by which micro-scraps fell from the MTPA surface.

When micro-scraps are produced, they are subjected to gravitational, frictional, and supporting forces. The relationships between the gravitational and frictional forces are shown in Equations (11) and (12):

(11) $$G_{x}=G\cdot cos\delta ,$$

(12) $$F_{f}=G\cdot sin\delta \cdot \mu ,$$

where *δ* is the angle between the surface that supports the micro-scraps and the vertical direction and *μ* represents the dynamic friction coefficient between the micro-scraps and the copper workpiece. If the condition *G_x_* > *F_f_* is satisfied, that is, if Equation (13) is satisfied, then the micro-scraps can slide off the sides of the MTPs [25,26], as shown in Figure 12:

(13) tanδ>μ

In this study, the angle between the surface that supports the micro-scraps and the vertical direction, *δ*, that is, the angle between the side and the bottom of an MTP, was 54.8°. The dynamic friction coefficient, *μ*, was 0.4; this value was obtained with a multi-function micro-friction test machine (UMT2). *δ* and *μ* satisfied Equation (13). Therefore, the fly-cutting method could effectively remove the micro-scraps from the workpiece surface when the working surface had a vertical orientation.

### 4.3. Diamond Tool Wear

**Figure 13:**
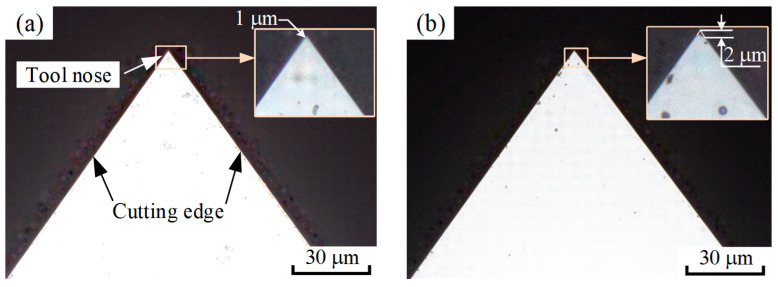
CLSM comparison of diamond tool profiles: (**a**) before fly-cutting and (**b**) after fly-cutting.

The experimental results show that, before machining, the profile of the cutting edge of the diamond tool was straight, and that the arc radius of the tool nose was 1 μm, as shown in Figure 13a. After machining, the cutting edge of the tool remained unbroken; it was also observed that the tool nose sustained damage 2 μm deep, as shown in Figure 13b. Compared to a horizontally oriented working surface [7], when the fly-cutting method was used with a vertically oriented working surface, there were no residual micro-scraps or side scratches on the MTP surfaces, the diamond tool nose sustained less damage, and the cutting edge experienced no breakage. When a working surface is vertically oriented, gravity and compressed air cause the micro-scraps to fall easily from the working surface, after which they are collected by the air suction device. Thus, very few micro-scraps on the workpiece surface are rubbed or squeezed by the tip or edge of the diamond tool. As a result, damage to the tip and edge of the diamond tools can be reduced, the tool life can be greatly improved, and a single diamond tool can process more MTPAs.

### 4.4. Residual Burrs

**Figure 14:**
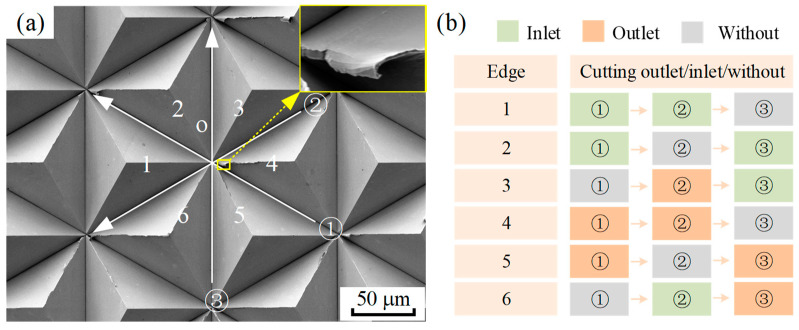
SEM results for the MTPA. (**a**) Six edges intersected at point o. The top right corner shows the residual burr of edge 4. (**b**) Analysis of the formation processes of the six edges.

Residual burrs can affect the optical functions of precision components. SEM analysis results obtained during this study showed that there were no burrs on the sides, tops, or cutting inlet edges of the MTPs, while a few burrs were present at the bottoms of the cutting outlet edges, as shown in Figure 14a. The analysis procedure is discussed next. In each sampling area, six edges intersected at point o, and these edges were numbered sequentially from 1 to 6. Edge 1 was formed by the inlet of the V-shaped groove cut along direction ① and the inlet of the V-shaped groove cut along direction ②. The V-shaped groove cut along direction ③ did not participate in the formation of edge 1, and edge 1 had no burrs. Edge 2 was formed by the inlet of the V-shaped groove cut along direction ① and the inlet of the V-shaped groove cut along direction ③. The V-shaped groove cut along direction ② did not participate in the formation of edge 2, and edge 2 had no burrs. Edge 3 was formed by the outlet of the V-shaped groove cut along direction ② and the inlet of the V-shaped groove cut along direction ③. The V-shaped groove cut along direction ③ did not participate in the formation of edge 3, and edge 3 had no burrs. Edge 4 was formed by the outlet of the V-shaped groove cut along direction ① and the outlet of the V-shaped groove cut along direction ②. The V-shaped groove cut along direction ③ did not participate in the formation of edge 4, and there were a few burrs on edge 4. Edge 5 was formed by the outlet of the V-shaped groove cut along direction ① and the outlet of the V-shaped groove cut along direction ③. The V-shaped groove cut along direction ② did not participate in the formation of edge 5, and there were a few burrs on edge 5. Edge 6 was formed by the inlet of the V-shaped groove cut along direction ② and the outlet of the V-shaped groove cut along direction ③. The V-shaped groove cut along direction ① did not participate in the formation of edge 6, and there were a few burrs on edge 6, as shown in Figure 14b. Therefore, 50% of the edges had no burrs, while 50% of the edges had a few burrs. The edges with a few burrs were all cutting outlet edges. The burrs on the cutting outlet edges could be removed by increasing the cutting speed or by performing repeated cutting.

## 5. Conclusions

To remove micro-scraps rapidly, a fly-cutting method to produce MTPAs on vertically oriented working surfaces was developed, and theoretical analyses and experimental research were conducted. The study produced three main conclusions:

- An MTPA produced by fly cutting when the working surface was vertically oriented had a clearly outlined structure, high dimensional accuracy, and a low surface roughness. Thus, fly-cutting machining of a vertical working surface is suitable for producing micro-structured arrays.
- There was no micro-scrap residue on the MTPA surface, the cutting edge of the diamond tool was not damaged, and the damage sustained by the tool nose was small. Thus, the vertical fly-cutting method effectively solves the problem of micro-scrap residue and prolongs the tool service life.
- Of six intersecting edges, 50% had no burrs and 50% had a few burrs. The edges with a few burrs were all cutting outlet edges. Edge burrs affect the edge straightness, making it necessary to reduce or eliminate them.
- The orthogonal experiment method was used to optimize the speed, feed speed, cutting depth, and cooling mode to reduce or eliminate the residual burrs on the edge, which will be the focus of future research.

## Figures and Tables

**Table 1:** Cutting parameters.

Rotational Speed, *n*	Feed Rate, *F*	Feed Amount, *f*	Actual Maximum Cutting Depth, *a_pm_*
1000 r/min	60 mm/min	60 µm/r	2 µm

**Table 2:** MTP size and surface roughness measurement results.

Measurement Area	Bottom Triangle Side Length, *l*		Angle between Opposite Sides, *β*		Edge Straightness, *A*/μm	Surface Roughness, *R_Z_*/nm
	Mean Value/μm	Deviation	Mean Value/°	Deviation		
1	114.44	0.89%	70.6	0.14%	2.2	19.0
2	115.10	0.32%	70.7	0.28%	1.8	18.8
3	115.04	0.37%	70.5	0.00%	3.1	19.4
Overall mean	114.86	0.53%	70.6	0.14%	2.37	19.1

## Data Availability

The original contributions presented in the study are included in the article, further inquiries can be directed to the corresponding authors.
